# Complex multitrophic species interactions and fitness costs: Intricate consequences of jasmonate and salicylate induced plant defences

**DOI:** 10.1111/1365-2656.70243

**Published:** 2026-04-14

**Authors:** Mônica F. Kersch‐Becker, Guilherme P. Pinheiro, Thaís S. Schweller, Martin Pareja, Victoria M. Pocius, José R. Trigo, Gustavo Q. Romero

**Affiliations:** ^1^ Department of Entomology, Center for Chemical Ecology, Center for Pollinator Research, Insect Biodiversity Center Pennsylvania State University University Park Pennsylvania USA; ^2^ Programa de Pós‐Graduação em Biologia Vegetal Universidade Estadual de Campinas (UNICAMP) Campinas São Paulo Brazil; ^3^ Laboratório de Interações Multitróficas e Biodiversidade, Departamento de Biologia Animal Universidade Estadual de Campinas (UNICAMP) Campinas São Paulo Brazil; ^4^ Departamento de Biologia Animal Universidade Estadual de Campinas (UNICAMP) Campinas São Paulo Brazil; ^5^ Douglas Products Liberty Missouri USA

**Keywords:** facultative mutualism, glandular trichomes, induced defences, jasmonate/salicylate tradeoffs, multitrophic interactions, plant resistance, pyrrolizidine alkaloids

## Abstract

1. Induced plant defences regulated by jasmonic acid (JA) and salicylic acid (SA) initiate a cascade of effects, from defence expression to changes in arthropod community structure and species interactions that can ultimately influence plant fitness. Yet the long‐term consequences of these processes remain poorly understood, particularly in perennial tropical systems.
2. We investigated how activation of JA and SA signalling pathways shapes arthropod community composition and plant reproductive success in *Trichogoniopsis adenantha* (Asteraceae), a glandular trichome‐bearing shrub native to the Brazilian Atlantic rainforest. Using chemical elicitors, we induced JA and SA defences for up to 16 months and monitored herbivore and predator assemblages, herbivory patterns, trichome expression, pyrrolizidine alkaloid concentrations and plant short‐term fecundity.
3. JA‐elicited plants exhibited higher pyrrolizidine alkaloid levels in flowers, reduced leaf herbivory and lower seed damage by pre‐dispersal seed predators. However, these plants experienced increased colonization by sap‐feeding insects. In contrast, SA‐elicited plants were more vulnerable to chewing herbivores but more resistant to sap‐feeders. Despite similar reductions in seed predation across both treatments, JA and SA induction significantly reduced ovary fertilization, likely due to changes in floral traits and decreased pollinator visitation, ultimately imposing substantial fitness costs. Additionally, seasonal turnover in arthropod communities was accompanied by temporal variation in how defences structured species composition and abundance.
4. Our findings demonstrate that phytohormone‐mediated defences can simultaneously induce resistance and susceptibility across herbivore guilds, with divergent impacts on predator associations. Although defence induction reduced herbivore damage, it compromised short‐term fecundity, revealing trade‐offs between defence and reproduction. By linking induced defences to multitrophic interactions over time, this study highlights the ecological complexity of plant defence strategies in biodiverse tropical ecosystems.

## Graphical abstract

This study reveals how long‐term activation of jasmonic and salicylic acid signalling reshapes arthropod communities and plant fitness across seasons. By showing that induced defences generate contrasting outcomes and cascading trade‐offs across trophic levels, it challenges the assumption that induced resistance is uniformly beneficial in natural systems.

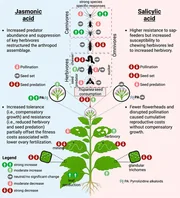

## Resumo

1. Defesas induzidas em plantas, reguladas pelos fitohormônios ácido jasmônico e ácido salicílico, desencadeiam efeitos em cascata que vão da expressão de defesas até mudanças na estrutura das comunidades de artrópodes e nas interações entre espécies, com consequências para a aptidão das plantas. No entanto, as implicações de longo prazo desses processos permanecem pouco compreendidas, especialmente em sistemas tropicais.
2. Investigamos como a ativação das rotas dos fitohormônios ácido jasmônico (AJ) e ácido salicílico (AS) afeta a composição da comunidade de artrópodes e o sucesso reprodutivo de *Trichogoniopsis adenantha* (Asteraceae), um arbusto nativo da Mata Atlântica brasileira com tricomas glandulares. Utilizando estimuladores químicos, induzimos defesas mediadas por AJ e AS ao longo de 16 meses e monitoramos as assembleias de herbívoros e predadores, os padrões de herbivoria foliar, a densidade de tricomas, as concentrações de alcaloides pirrolizidínicos e a fecundidade das plantas.
3. Plantas estimuladas com ácido jasmônico apresentaram maiores concentrações de alcalóides pirrolizidínicos tanto em folhas quanto em flores, menor herbivoria foliar e menor dano às sementes. No entanto, essas plantas exibiram maior colonização por insetos sugadores. Em contraste, plantas estimuladas com ácido salicílico foram mais suscetíveis a herbívoros mastigadores, porém mais resistentes a insetos sugadores. Apesar de reduções semelhantes na predação de sementes em ambos os tratamentos, a indução por ácido jasmônico e ácido salicílico reduziu significativamente a fertilização dos ovários de *T. adenantha*, provavelmente em decorrência de alterações em características florais e da diminuição da visitação por polinizadores, impondo custos substanciais à aptidão das plantas. Além disso, os efeitos das defesas sobre a estrutura das comunidades de artrópodes variaram ao longo do tempo, com impactos mais pronunciados durante a estação seca.
4. Nossos resultados demonstram que defesas mediadas por fitohormônios podem induzir simultaneamente resistência e suscetibilidade entre diferentes guildas de herbívoros, gerando impactos contrastantes nas associações com predadores. Embora a indução de defesas tenha reduzido o dano causado por herbívoros, ela comprometeu a fecundidade, evidenciando trade‐offs entre defesa e reprodução. Ao integrar defesas induzidas a interações multitróficas ao longo do tempo, este estudo destaca a complexidade ecológica das estratégias de defesa das plantas em ecossistemas tropicais altamente biodiversos.

## 1 INTRODUCTION

Plants defend themselves against herbivores using a wide array of traits that vary in timing, expression and function. Direct defences, such as toxic, anti‐digestive and repellent compounds, interfere with herbivore feeding and development (Kessler, 2015; Kessler & Kalske, 2018). Plants also deploy indirect defences that recruit and retain natural enemies of herbivores, including herbivore‐induced plant volatiles, nutritional rewards and physical structures that provide shelter (Aljbory & Chen, 2018; Turlings & Erb, 2018). Defensive traits are not only diverse but also plastic, activated in response to herbivory. Over the past three decades, induced resistance has emerged as a critical strategy by which plants minimize metabolic costs, deploying defences only when under attack (Karban, 2020).

Plants activate these inducible defences through phytohormone‐mediated signalling pathways, primarily involving salicylic acid (SA) and jasmonic acid (JA) (Monte, 2023). These phytohormones regulate defence responses to different attackers, with JA signalling typically enhancing resistance to chewing herbivores via toxic secondary metabolites, digestibility reducers and trichomes, and SA mediating resistance to sap‐sucking herbivores and pathogens through pathogenesis‐related proteins and systemic acquired resistance (Gasperini & Howe, 2024; Klessig et al., 2018). Although JA and SA signalling were traditionally viewed as functionally distinct, growing evidence reveals extensive crosstalk that can generate synergistic or antagonistic effects depending on ecological context (Aerts et al., 2021; Kersch‐Becker et al., 2017; Schweiger et al., 2014; Thaler et al., 2012). This plasticity can impose costs, as signalling crosstalk may reallocate resources and inadvertently increase vulnerability to subsequent attacks (Schweiger et al., 2014), with consequences that propagate across herbivores and higher trophic levels (Kersch‐Becker et al., 2017).

In natural and perennial systems, phytohormone‐mediated defences operate within arthropod communities that change seasonally, altering herbivore pressure, natural enemy dynamics and the expression of induced resistance over time (Calixto et al., 2021; Karolewski et al., 2013; Van Zandt & Agrawal, 2004). Consequently, short‐term experiments often fail to capture how defence induction influences longer‐term growth and reproductive outcomes, including defence–growth–reproduction trade‐offs that unfold across seasons (Skovmand et al., 2024; van Dijk et al., 2024). This limitation is especially consequential for obligate outcrossing plants, where defensive traits can simultaneously influence herbivores, pollinators and seed set through community‐wide cascades (Lucas‐Barbosa, 2016; Rusman et al., 2018). These gaps highlight the need for community‐level studies that capture how ecological complexity and temporal variation shape the outcomes of phytohormone‐mediated defences.

In a manipulative field experiment spanning more than one year, we tested how jasmonic acid (JA) and salicylic acid (SA) elicitation structure arthropod communities and influence plant reproduction across seasons. We repeatedly applied chemical elicitors to prime the JA and SA pathways in *Trichogoniopsis adenantha* (DC.) R.M.King & H.Rob. (Asteraceae), a glandular trichome‐bearing species that produces toxic pyrrolizidine alkaloids and engages in facultative mutualism with flower‐dwelling spiders (Kersch‐Becker et al., 2018b; Morais‐Filho & Romero, 2010; Romero et al., 2008). We hypothesized that JA and SA elicitation would differentially alter plant defensive traits, arthropod community composition and plant reproduction, with season‐dependent effects that intensify during periods of high herbivore pressure. Because investment in induced chemical and structural defences draws on finite plant resources, we predicted a trade‐off in which increased allocation to defences reduces vegetative growth and floral production while simultaneously limiting damage from herbivores. Specifically, we expected JA‐elicited plants to exhibit higher pyrrolizidine alkaloid concentrations and trichome density, leading to reduced leaf damage and lower abundance of chewing herbivores, including pre‐dispersal seed predators. In contrast, we expected SA‐elicited plants to host fewer phloem‐feeding herbivores. We further predicted that reduced herbivore pressure would cascade to higher trophic levels, increasing the abundance of omnivorous and carnivorous arthropods on elicited plants via enhanced herbivore‐induced plant volatile emission. With respect to plant performance, we predicted that defence induction would reduce vegetative growth and/or flower production relative to control plants, but that these costs would be offset by reduced herbivory and seed predation, resulting in greater seed production and overall reproductive success. Together, these predictions allowed us to test whether phytohormone‐mediated defence induction increases plant fecundity by reducing herbivory, or whether allocation costs constrain growth and reproduction, revealing trade‐offs among defence, growth and reproductive investment.

## 2 METHODS

### 2.1 Study area

We conducted the experiments over a 16‐month period, from April 2015 to August 2016, at the Serra do Japi Ecological Reserve, Jundiaí, São Paulo, Brazil (23°11′ S, 46°52′ W). This forest is characterized as a semi‐deciduous forest within the Atlantic Forest biome. The region experiences a distinct seasonal climate, with a warm, rainy season from November to April and a cooler, dry season from June to September. Mean monthly temperatures range from 13.5°C in July to 20.3°C in January.

### 2.2 Study system

*Trichogoniopsis adenantha* is a small, perennial shrub native to southeastern Brazil and abundant along forest edges in the Atlantic Forest. It interacts with a diverse arthropod community and expresses multiple defensive traits that deter herbivores and enhance reproductive success. Leaves and stems bear non‐glandular and glandular trichomes that exude an adhesive substance capable of trapping small insects (Romero et al., 2008). These glandular trichomes mediate facultative mutualistic interactions with spiders, including the crab spider *Misumenops argenteus* and the lynx spiders *Peucetia flava* and *Peucetia rubrolineata*, which reduce herbivore oviposition and remove larvae, substantially increasing plant fitness (Kersch‐Becker et al., 2018b; Romero et al., 2008; Romero & Vasconcellos‐Neto, 2004). In addition to its physical and biotic defences, *T. adenantha*'s vegetative and reproductive tissues contain pyrrolizidine alkaloids (PA) that deter generalist herbivores while attracting certain specialist species (Trigo, 2011). Flowering peaks during the rainy‐wet season (January–April), with each branch producing approximately seven flowerheads, each containing around 40 florets. As an obligate outcrosser, *T. adenantha* depends on pollinators for fertilization, attracting a diverse assemblage of butterflies, moths, bees and flies (Rezende et al., 2025). Herbivores include chewing caterpillars and sap‐feeding insects, while flowers and developing seeds are frequently attacked by lepidopteran and dipteran larvae that are themselves parasitized by parasitoid wasps, adding further trophic complexity to this specialized plant–arthropod network (Kersch‐Becker et al., 2018b; Romero et al., 2008; Romero & Vasconcellos‐Neto, 2004, 2005).

### 2.3 Greenhouse experiment

To test whether jasmonic acid‐ or salicylic acid‐mediated defence elicitation increases pyrrolizidine alkaloid (PA) content and glandular and non‐glandular trichome production, we conducted a greenhouse experiment. In March 2015, we collected *T. adenantha* seedlings from the Serra do Japi Ecological Reserve and transplanted them into 25 cm diameter pots containing a mixture of natural and commercial soil. Plants were maintained in a greenhouse at the University of Campinas. In July 2015, when plants began flowering, we randomly assigned each plant to one of three treatments: (i) untreated controls, (ii) salicylic acid (SA)–elicited plants treated with benzo(1,2,3)thiadiazole‐7‐carbothioic acid *S*‐methyl ester (BTH), and (iii) jasmonic acid (JA)–elicited plants treated with methyl jasmonate (MeJA). To elicit the SA pathway, we prepared a 1.2 mmol L^−1^ BTH solution. To elicit the JA pathway, we prepared a 1.5 mmol L^−1^ MeJA solution by dissolving 20 μL of pure MeJA in 750 μL ethanol and diluting the mixture with distilled water. Both SA‐elicited and control plants received 750 μL of ethanol. Concentrations were selected based on previous studies demonstrating effective defence priming without phytotoxicity or strong antagonistic signalling crosstalk (Mur et al., 2006; Subedi et al., 2025; Thaler et al., 1996, 1999).

#### 2.3.1 Pyrrolizidine alkaloid content

Five days after treatment application, we sampled 24 plants and collected ca. 300 mg of senescent, mature and young leaves, as well as inflorescence buds and open inflorescences, to assess pyrrolizidine alkaloid (PA) content. To extract and quantify PAs, we used a colorimetric method following established protocols (Trigo et al., 1993). For additional details, see Supporting Information (S1). Because defence induction can be tissue specific, we analysed each tissue type separately using generalized linear mixed‐effects models (GLMMs) with a Cauchy error distribution using the Generalized Regression platform in JMP, including treatment and number of leaves as fixed effects in JMP 18.

#### 2.3.2 Trichome density

We quantified the number of glandular and non‐glandular trichomes on both the abaxial and adaxial surfaces of leaves using a stereomicroscope. For each plant, we collected two leaf discs (0.5 cm diameter) from the first leaf produced after treatment application. We summed the trichomes from the two discs to obtain a single value per plant. In total, we sampled 48 plants, 16 replicates per treatment. We analysed trichome density using GLMs with a negative binomial distribution, including plant treatment as a fixed effect.

### 2.4 Field experiment

#### 2.4.1 Setup and experimental design

We carried out three independent trials initiated at different times: Trial 1 (April 2015 to July 2016), Trial 2 (January 2016 to August 2016), Trial 3 (February 2016 to August 2016). Due to plant mortality during trial 1, additional plants were selected to establish trials 2 and 3; because these plants were selected later, the duration of trials 2 and 3 was shorter. At the start of each trial, we selected 39, 15 and 15 undamaged *T. adenantha* seedlings, respectively. Seedlings averaged 43 ± 1.7 cm in height, with no significant differences at the start of the experiment (*F*_2,62_ = 1.53, *p* = 0.226). In total, 23 single‐plant replicates per treatment were distributed across trials. Plants were grouped into spatial blocks based on proximity. Within a block, we selected plants that were at least 1 m apart, maintaining a minimum distance of 3 m between blocks. We randomly assigned each plant within a block to one of three treatments: (i) untreated control, (ii) SA‐elicited, or (iii) JA‐elicited. We reapplied the treatment weekly for the duration of the experiment. Sampling was authorized by the Fundação Serra do Japi (Authorization 005/2016, 002/2015, 010/2015) and the Instituto Chico Mendes de Conservação da Biodiversidade (SISBIO licence #47340‐1 and #47338‐2, #47338‐1) and all sampled species were registered in the Sistema Nacional de Gestão do Patrimônio Genético e do Conhecimento Tradicional Associado (SISGEN registry number A6BB868). Our work did not require any ethics approvals.

#### 2.4.2 Plant growth, phenology and herbivory assessment

We monitored plant growth and phenology monthly over the course of 16, 8 or 6 months for trials 1, 2 and 3, respectively. We measured plant height to the nearest centimetre and analysed final height (log‐transformed) using a generalized linear mixed‐effects model (GLMM) with Gaussian distribution. The model included treatment, initial plant height, trial and number of days alive as fixed effects, and block as a random effect. We included the number of days alive as a covariate in our models to account for plant mortality. To monitor leaf production and loss, we tied a yarn marker on the apical meristem of each plant prior to the start of the experiment. This marker allowed us to track leaf production above the yarn and assess leaf loss over time. We counted newly produced leaves once a month. To estimate leaf loss, we compared the number of leaves at time *t* (excluding newly produced leaves) with the count at *t* − 1 (the previous census). To assess treatment effects on plant survival, we used survival analysis with Cox's proportional hazard model. We analysed the number of leaves produced and lost using GLMMs with a negative binomial error distribution, including treatment, trial and days alive as fixed effects and block as a random effect. We quantified foliar herbivory monthly by visually assigning each leaf to one of six damage categories: 0%, <5%, <25%, <50%, <75%, <99%. For each plant in each month, we calculated percentage herbivory as a weighted average by multiplying the number of leaves in each damage category by the category endpoint percentage and dividing by the total number of leaves. These monthly herbivory estimates were then averaged across all survey months for each plant to obtain a single experiment‐wide estimate of mean foliar herbivory. We analysed herbivory as a proportion by scaling per cent leaf damage to the interval 0–1 and applying an arcsine square‐root transformation to stabilize variance. The transformed data were analysed using a GLMM with a Gamma error distribution, including treatment, trial and days alive as fixed effects and block as a random effect.

#### 2.4.3 Arthropod community and trophic guilds

We performed visual arthropod censuses weekly, observing each plant for 1 min to record the abundance of arthropods by morphospecies. We collected a voucher specimen of each morphospecies for later identification. In the laboratory, we identified each specimen to the lowest possible taxonomic level. To investigate the effects of elicitation on different arthropod groups, we categorized the arthropod community into feeding guilds. Herbivores were classified as chewers, sap‐suckers, miners, or flowerhead endophages (pre‐dispersal seed predators). Carnivores included both predators and parasitoids, while omnivores comprised species known to feed on both plants and insects. We based these classifications on direct observations and natural history records. We also analysed the most abundant and ecologically relevant species within each feeding guild. We stored all specimens in the *Laboratório de Interações Multitróficas e Biodiversidade* at the Universidade Estadual de Campinas. In total, we conducted 56 arthropod censuses during trial 1, 26 during trial 2 and 18 during trial 3.

To evaluate whether treatments influenced the composition of arthropod communities associated with *T. adenantha*, we conducted permutational multivariate analysis of variance (PERMANOVA) based on Bray–Curtis dissimilarity. Analyses were performed on presence/absence data for each arthropod morphospecies, excluding morphospecies represented by a single individual across all samples. Because the data were binary, Bray–Curtis dissimilarities were calculated with the binary = TRUE option in the vegan package, which is equivalent to Sørensen dissimilarity for presence–absence data. The PERMANOVA model tested the marginal effect of treatment while accounting for log‐transformed days alive and plant height as covariates. Permutations (9999) were restricted within trial to control for trial‐level variation. We then conducted pairwise PERMANOVAs to evaluate differences in species composition among treatments. All multivariate analyses were performed in R using the *vegan* package. To analyse cumulative arthropod community metrics, including total abundance, species richness, Shannon diversity and the abundance of specific species or feeding guilds, we used either GLM or GLMMs with Poisson, zero‐inflated Poisson, negative binomial or zero‐inflated negative binomial error distributions, depending on the response variable (see Table S1 for distribution details). We included the number of days alive as a covariate to account for mortality. To examine whether the effects of defence elicitation varied seasonally, arthropod abundances were summed separately for the dry and wet seasons. Temporal patterns in arthropod abundance were analysed using GLMMs including plant elicitation treatment, season and their interaction, and trial as fixed effects. Trial was included as a fixed effect in most models, but was modelled as a random effect when this structure improved model fit based on model comparison. Plant identity was included as a random effect to account for repeated measurements on the same plant over time, whereas block was excluded because it did not improve model fit. Models were fit using negative binomial or zero‐inflated negative binomial error distributions, as appropriate (see Table S1), with post hoc tests used to compare elicitation treatments within each season. We conducted these analyses using the *glmmTMB* package in R.

#### 2.4.4 Plant fitness and seed damage

We collected the flowerheads over the course of the experiment once they reached the pre‐dispersal stage, when fertilized ovaries would have developed into mature achenes and pre‐dispersal seed predator larvae would have reached their final developmental stages. We placed each flowerhead into a separate gelatin capsule and monitored them until either pre‐dispersal seed predators or their parasitoids emerged. Then, we dissected each flowerhead and counted the number of fertilized and unfertilized ovaries and recorded whether each ovary was undamaged or damaged. We identified pre‐dispersal seed predators based on their pupae and the characteristic damage they inflicted. For each flowerhead, we documented both the identity and abundance of pre‐dispersal seed predators and their associated parasitoids.

To determine whether plant defence elicitation affect flowerhead production, we used a GLM with negative binomial distribution. The model included treatment, trial and days alive as fixed effects. To assess effects of elicitation on the number of ovaries per flowerhead, we calculated the average number of ovaries per flowerhead (total ovaries divided by the number of flowerheads) and analysed this response using a GLM with Gaussian distribution, including treatment and trial as fixed effects. We used total number of intact seeds produced per plant as a proxy for plant fecundity and analysed this variable using a zero‐inflated negative binomial GLM, with treatment, trial and days alive as fixed effects. We used GLMs with a Poisson error distribution to analyse the abundance of pre‐dispersal seed predators focal taxa: *Trupanea* sp. (Diptera: Tephritidae), Geometridae sp. (Lepidoptera) and *Melanagromyza* sp. (Diptera: Agromyzidae). These models included plant defence treatment and the number of flowerheads as fixed effects. We used GLMs with a binomial error distribution and logit link function to model reproductive and damage outcomes, specifying responses as counts of successes with corresponding numbers of trials. Pollination success was modelled as the probability of ovary fertilization (fertilized ovaries relative to total ovaries per flowerhead), and ovary damage as the probability that an ovary was damaged (damaged ovaries relative to total ovaries per plant), with ovary counts summed at the plant level. Pre‐dispersal seed predation by *Trupanea* sp. was modelled as the probability of ovary consumption within infested flowerheads (ovaries consumed by larva relative to total ovaries per flowerhead). We restricted analyses to *Trupanea*‐damage flowerheads because other seed predators occurred at low frequencies. We also modelled the probability of *Trupanea* larval parasitism using GLMs with binomial error distribution, specifying the response as the number of parasitized larvae relative to the total number of *Trupanea* larvae per plant; analyses were restricted to *Trupanea* because no other endophagous taxa were parasitized. All binomial models included plant defence treatment and flowerhead number as fixed effects.

We tested whether plant defence elicitation influences fecundity through increased resistance to pre‐dispersal seed predators or through pollination‐related processes. Specifically, we examined whether plant defence treatment and flowerhead condition (damaged vs. undamaged) affected the probability of producing viable seeds given total ovary production. We used binomial GLMs with a logit link to model the probability of producing viable seeds as a function of plant defence treatment, flowerhead condition, their interaction and trial. Plant‐level estimates were obtained by averaging ovary number per flowerhead. Although flowerhead damage was not experimentally manipulated, this approach allows us to evaluate whether associations among defence elicitation, flowerhead damage and seed set are consistent with both damage‐related and damage‐independent pathways linking plant defences to reproductive output. A treatment effect indicates defence‐mediated changes in seed set independent of damage (e.g. pollination effects), a flowerhead condition effect indicates direct effects of herbivory, and a significant interaction indicates that plant defences influence seed set through both resistance and pollination.

#### 2.4.5 General statistical approach

We used the Shapiro‐Wilks test to detect departure from normality. We evaluated the fit and assumptions of our generalized linear model using the DHARMa package in R (Hartig, 2016). To assess model residuals and identify potential deviations from expected distributions, we simulated standardized residuals with *simulateResiduals*() using the fitted model. We visually inspected the resulting diagnostic plots to assess residual distribution, detect potential outliers and evaluate overall model fit. Table S1 provides an overview of the models used to analyse each response variable.

## 3 RESULTS

### 3.1 Pyrrolizidine alkaloid content

Defence elicitation affected pyrrolizidine alkaloid (PA) concentrations in a tissue‐specific manner (Table S1, Figure 1A). In reproductive tissues, JA elicitation increased PA concentrations by 103% in buds and 34% in open flowerheads compared to control plants, whereas SA elicitation did not differ from controls (Bud: *χ* ^2^_2_ = 7.97, *p* = 0.019; Open flowerheads: *χ* ^2^_2_ = 15.10, *p* < 0.001). In young leaves, elicitation had no effect on PA concentrations (*χ* ^2^_2_ = 0.15, *p* = 0.928). In mature leaves, JA‐elicited plants exhibited 106% higher concentrations than SA‐elicited plants (*χ* ^2^_2_ = 40.58, *p* < 0.001). In contrast, senescent leaves showed significant variation in PA concentrations in response to treatments (*χ* ^2^_2_ = 6.07, *p* = 0.048); however, PA concentrations were low, and pairwise Tukey comparisons did not detect differences among treatments.

**FIGURE 1:**
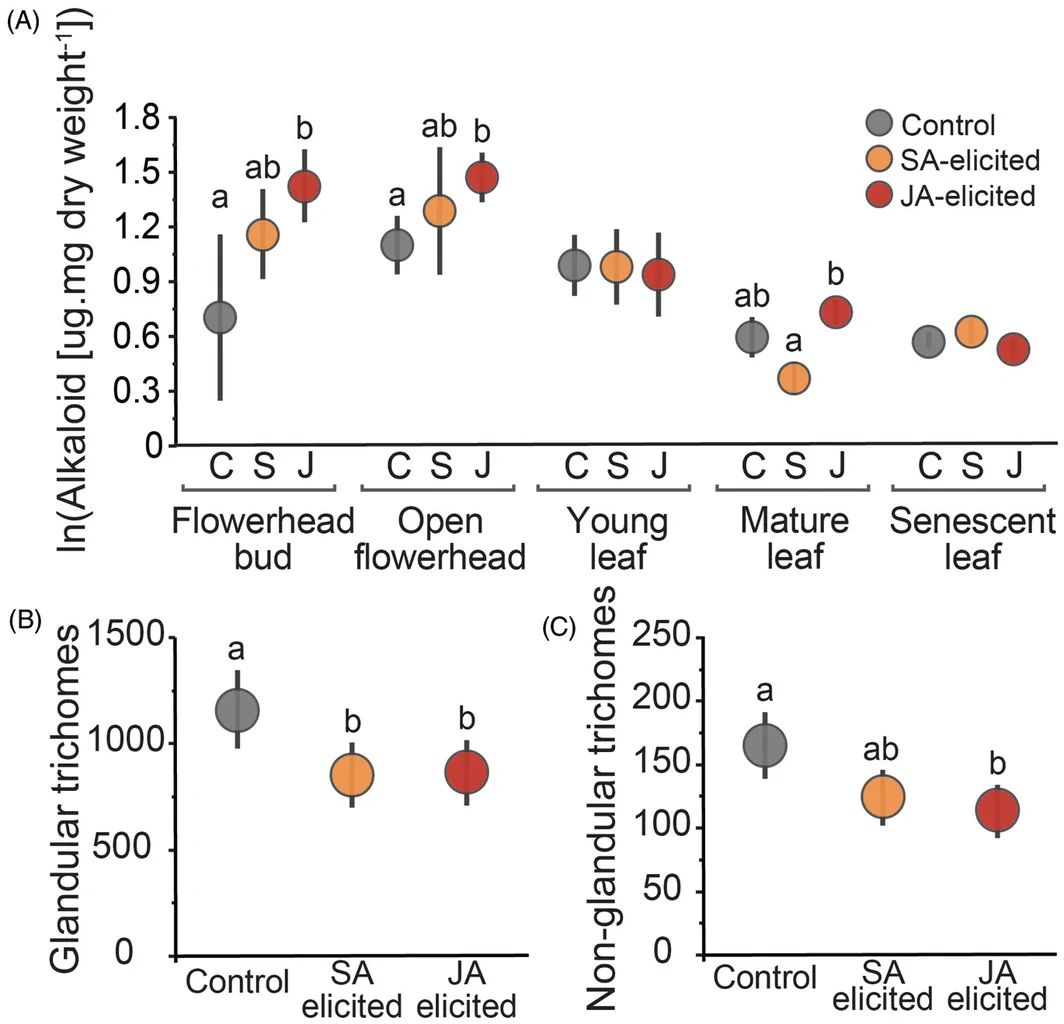
Effects of plant defence elicitation on pyrrolizidine alkaloid content and trichome density in *T. adenantha*. Panels show estimated marginal means (±95% CI) of (A) pyrrolizidine alkaloid content (ln‐transformed; μg mg^−1^ dry weight) in reproductive (buds and open flowerheads) and vegetative tissues (young, mature and senescent leaves), (B) glandular trichomes density per 1 cm^2^, (C) simple trichomes density per 1 cm^2^. Plant defence treatments are indicated by colour: Untreated control plants (grey), salicylic acid–elicited plants (SA; orange) and jasmonic acid–elicited plants (JA; red). Different letters above bars indicate significant differences among treatments (*p* < 0.05, Tukey's HSD post hoc test). In panel (A), lowercase letters denote treatment differences within each tissue categories.

### 3.2 Trichome density

JA and SA elicitation reduced glandular trichome density, with control plants exhibiting 1.36‐fold higher densities than elicited plants (Glandular: *χ* ^2^_2_ = 8.97, *p* = 0.011, Figure 1B). In contrast, JA elicitation reduced non‐glandular trichome density, with control plants exhibiting 1.47‐fold higher density than JA‐treated plants, whereas SA did not differ from either JA or control plants (*χ* ^2^_2_ = 10.67, *p* = 0.005, Figure 1C).

### 3.3 Plant growth and herbivory

Plant mortality and final plant height did not differ among treatments (Survival: Wilcoxon *χ* ^2^_2_ = 0.94, *p* = 0.624; Height: *F*_2,61_ = 0.23, *p* = 0.795). Over the course of the experiment, JA‐elicited plants experienced 0.8‐fold greater leaf loss but also produced 1.38‐fold more leaves compared to control and SA‐elicited plants (Leaf loss *F*_2,40_ = 4.29, *p* = 0.020, Leaf production *F*_2,38_ = 4.57, *p* = 0.017, Figure 2A). JA‐elicited plants exhibited increased resistance, experiencing a 23% reduction in herbivory relative to control plants. In contrast, SA‐elicited plants showed increased susceptibility, with 31% greater leaf damage compared to JA‐elicited plants, but did not differ from control plants (*F*_2,44_ = 7.11, *p* = 0.002, Figure 2B).

**FIGURE 2:**
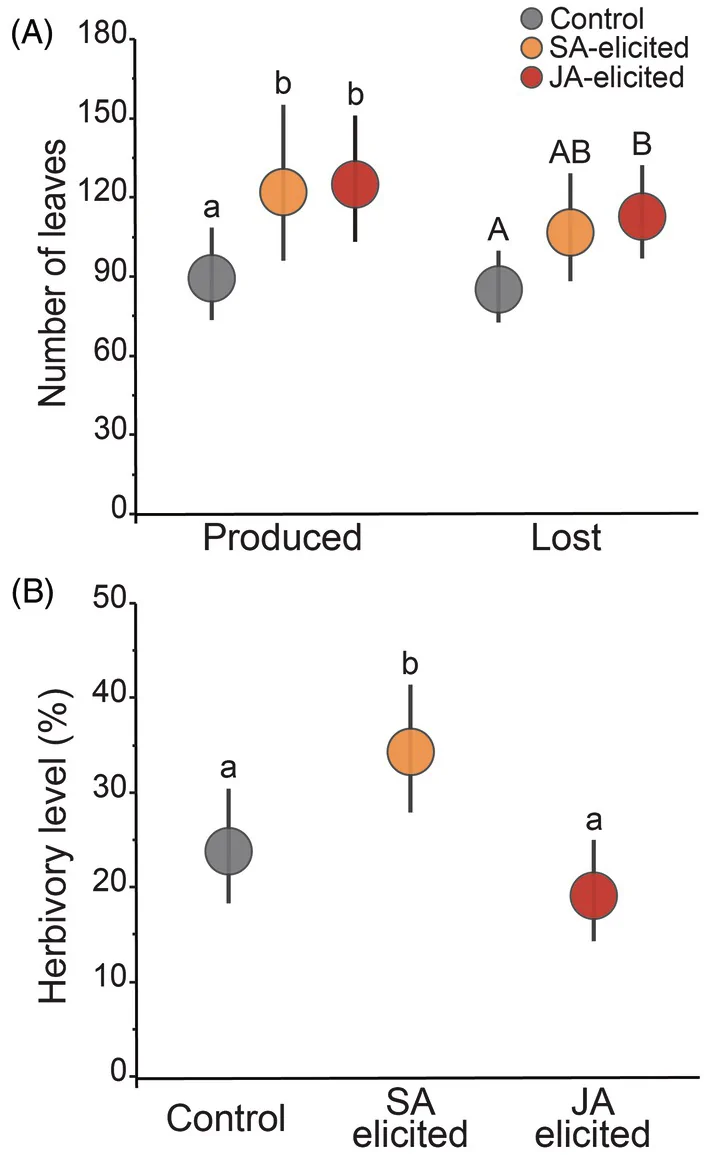
Effects of plant defence elicitation on leaf production, leaf loss and herbivory in *T. adenantha*. (A) Cumulative number of leaves produced and lost per plant over the course of the experiment. (B) Mean herbivory levels per plant. Plant defence treatments are indicated by colour: Untreated control (grey), salicylic acid–elicited plants (SA; orange) and jasmonic acid–elicited plants (JA; red). Bars show back‐transformed estimated marginal means (±95% CI). Different letters above bars indicate significant differences among treatments (*p* < 0.05, Tukey's HSD post hoc test). In panel (A), lowercase letters denote differences in leaf production, whereas uppercase letters denote differences in leaf loss.

### 3.4 Arthropod community and trophic guilds

Over the course of the experiment, we recorded a total of 14,048 individuals representing at least 78 morphospecies, with 99.9% classified into three trophic guilds: herbivores (13,428 individuals, 95.6%), carnivores (373 individuals, 2.7%) and omnivores (230 individuals, 1.6%). The most abundant herbivores recorded included whiteflies (Aleyrodidae: Hemiptera), leaf mining insects, leafhoppers (Hemiptera), geometrid caterpillars (Geometridae: Lepidoptera) and flowerhead endophages (Diptera and Lepidoptera). The dominant carnivores were damsel bugs (Nabidae) and spiders from the families Clubionidae, Oxyopidae, Thomisidae and Theritidae. The most abundant omnivores included ants (Formicidae) and mirid insects (Miridae). While SA and JA elicitation did not influence the total number of species recorded throughout the experiment (*χ* ^2^_2_ = 0.10, *p* = 0.608) or species diversity (*χ* ^2^_2_ = 4.40, *p* = 0.092), they resulted in significant differences in arthropod species composition on *T. adenantha* (*F*_2,62_ = 1.49, *p* = 0.044). SA‐ and JA‐elicited plants harboured distinct arthropod communities (*F*_2,40_ = 1.83, *p* = 0.022, Figure 3). In contrast, community composition did not differ significantly between control plants and either SA‐ or JA‐elicited plants (control vs. SA: *F*_2,40_ = 1.44, *p* = 0.1117; control vs. JA: *F*_2,42_ = 1.02, *p* = 0.384).

**FIGURE 3:**
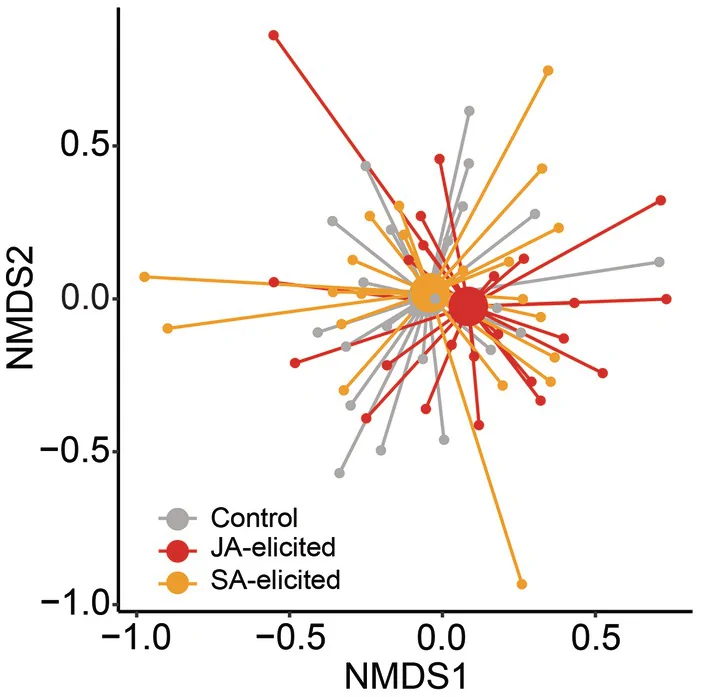
Non‐metric multidimensional scaling (NMDS) ordination of arthropod community composition associated with *T. adenantha* plants subjected to different defence elicitation treatments. Points represent individual plants and are coloured by treatment: Untreated controls (grey), jasmonic acid–elicited plants (JA; red) and salicylic acid–elicited plants (SA; orange). Lines connect individual samples to treatment centroids (mean community composition). Ordination is based on Bray–Curtis dissimilarities calculated from presence–absence data.

The abundance of whiteflies (Aleyrodidae sp.), the most abundant arthropod in our study (*N* = 12,438), strongly influenced the overall abundance of sap‐sucking herbivores. Therefore, we analysed the effects of plant defence elicitation on whiteflies and other sap‐sucking herbivores separately. Whitefly abundance was 1.82‐fold higher on JA‐elicited plants compared to both control and SA‐elicited plants, which exhibited similar whitefly densities (*χ* ^2^_2_ = 5.55, *p* = 0.06, Figure 4A). In contrast, plant defence elicitation did not significantly affect the abundance of other sap‐sucking herbivores (*χ* ^2^_2_ = 0.259, *p* = 0.878, Figure 4A). SA‐elicited plants exhibited a 0.13‐fold reduction in leaf‐mining insect abundance compared to control and JA‐elicited plants (*χ* ^2^_2_ = 6.48, *p* = 0.039, Figure 4B). JA‐elicited plants showed increased resistance to chewing herbivores, supporting a 0.62‐fold reduction in chewer abundance compared to SA‐elicited and control plants (*χ* ^2^_2_ = 7.04, *p* = 0.030, Figure 4C). Accordingly, JA‐elicited plants harboured a 0.27‐fold reduction in Geometridae sp. caterpillars, the most abundant leaf‐chewing herbivore, compared to SA‐elicited and control plants (*χ* ^2^_2_ = 7.004, *p* = 0.030, Figure 4C). On SA‐elicited plants, the abundance of chewing herbivores increased herbivory (*χ* ^2^ = 4.02, *p* = 0.045, Figure 4D). In contrast, JA‐elicited plants and control plants' chewer abundance reduced leaf herbivory (*χ* ^2^ = 4.35, *p* = 0.037; Control: *χ* ^2^_1_ = 6.96, *p* = 0.008, Figure 4D).

**FIGURE 4:**
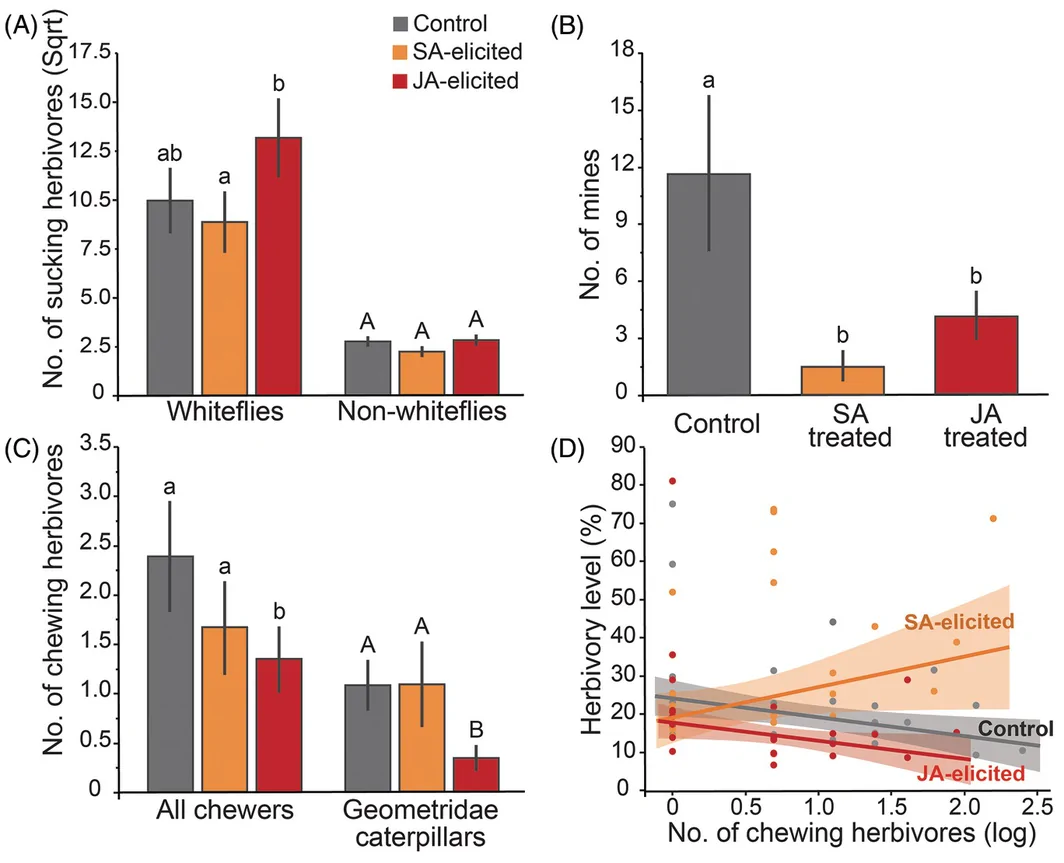
Arthropods abundance and herbivory responses to plant defence elicitation. (A–C) Bar graphs showing the mean cumulative abundance of arthropods recorded throughout the experiment by feeding guild: (A) whiteflies and other sap‐sucking herbivores, (B) leaf‐mining insects and (C) chewing herbivores, including Geometridae caterpillars. (D) Regression showing the relationship between the abundance of chewing herbivores and observed herbivory level across treatment; shaded areas represent 95% confidence intervals. Plant defence treatments are indicated by colour: Untreated controls (grey), SA‐elicited (orange) and JA‐elicited (red) plants. Bars represent ±1 standard error (SE). Different letters above each bar indicate significant differences among treatments (*p* < 0.05 following post hoc tests). In panel (A), lowercase letters compare the number of whiteflies, whereas uppercase letters denote differences in other sap‐sucking herbivores. In panel (C), lowercase letters denote differences in total chewers, whereas uppercase letters denote differences in Geometridae caterpillars.

The abundance of omnivores and carnivores was not affected by the defence treatments (Carnivores *χ* ^2^_2_ = 0.691, *p* = 0.708, Omnivores *χ* ^2^_2_ = 1.56, *p* = 0.460, Figure 5A,C). However, the true bug *Macrolophus aragarcanus*, the most abundant omnivore, was 2.5 times more abundant on control plants compared to SA‐elicited plants (*χ* ^2^_2_ = 7.31, *p* = 0.026, Figure 5B). Within the carnivore guild, important species‐specific responses were observed, which likely contributed to the lack of a significant overall effect. The most abundant carnivorous arthropods recorded in this study were the lynx spider *Peucetia rubrolineata*, the crab spider *Misumenops argenteus*, a Clubionidae spider, and a damsel bug (Nabidae sp.). While the abundance of *M. argenteus* was not affected by the elicitation treatments (*χ* ^2^_2_ = 2.49, *p* = 0.433), JA and SA elicitation had contrasting effects on other carnivore species. SA‐elicited plants exhibited a 0.26‐fold reduction in damsel bug (Nabidae sp.) abundance compared to JA‐elicited and control plants (*χ* ^2^_2_ = 8.24, *p* = 0.016, Figure 5D). In contrast, Clubionidae spiders were 2.33‐fold more abundant on JA‐elicited compared to control plants (*χ* ^2^_2_ = 6.38, *p* = 0.041, Figure 5E). The lynx spider *P. rubrolineata* was 10‐fold more abundant on SA‐elicited plants compared to JA‐elicited and control plants (*P. rubrolineata χ* ^2^_2_ = 9.27, *p* = 0.010, Figure 5F).

**FIGURE 5:**
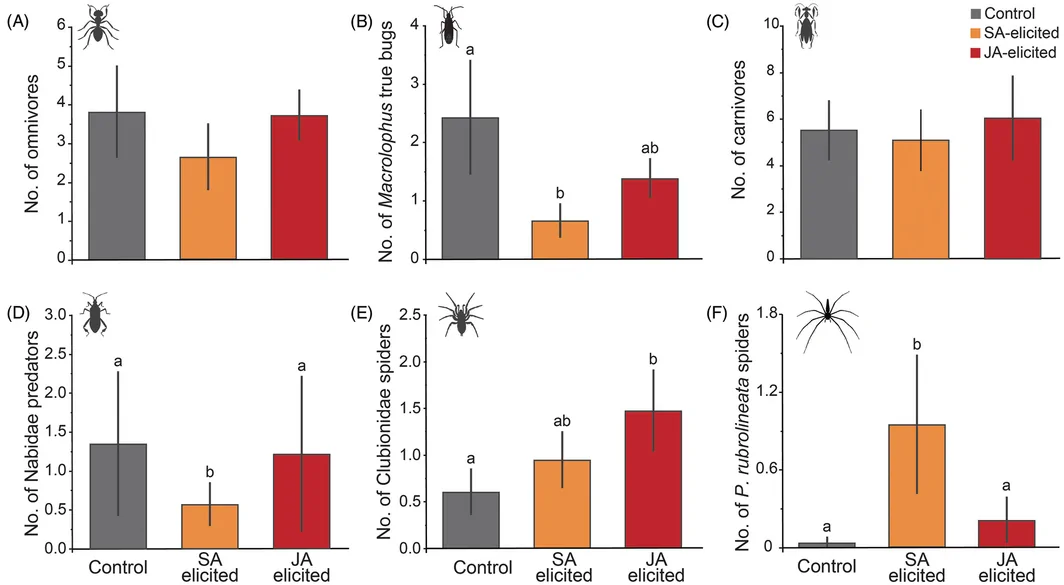
Cumulative abundance of omnivorous and carnivorous arthropods recorded throughout the experiment. (A) omnivores, (B) *Macrolophus aragarcanus* (Miridae) true bugs, (C) carnivores, and the most abundant carnivore taxa: (D) Nabidae damsel bugs, (E) Clubionidae spiders, and (F) lynx spider *Peucetia rubrolineata*. Plant defence treatments are indicated by colour: Untreated control plants (grey), salicylic acid–elicited plants (SA; orange) and jasmonic acid–elicited (JA; red) plants. Bars represent means ± 1 standard error (SE). Different letters above each bar indicate significant differences among treatments (*p* < 0.05, Tukey's HSD post hoc test).

### 3.5 Temporal variation

The arthropod community composition exhibited strong temporal variation that differed among plant defence treatment (Figures 6 and 7, Table S1). Plant defence elicitation did not alter species composition in the wet season (PERMANOVA: *F*_2,55_ = 0.61, *p* = 0.863), but significantly structured communities during the dry season (PERMANOVA: *F*_2,51_ = 2.41, *p* = 0.003). Consistent with these community‐level patterns, several dominant taxa showed pronounced treatment‐by‐season interactions. Chewing herbivore abundance varied across seasons in response to plant defence elicitation (*χ* ^2^_4_ = 12.84, *p* = 0.012, Figure 7A). JA elicitation markedly reduced chewer abundance during both dry seasons (82% in the first dry season and 62% in the second dry season relative to controls), while no defence elicitation effects were detected during the wet season. Similar results were observed for *Geometridae* sp., the most abundant chewing herbivore (Table S1, Figure 7B). Leaf‐mining insects responded to defence elicitation in a season‐dependent manner (*χ* ^2^_4_ = 13.47, *p* = 0.009, Figure 7C). SA‐elicited plants did not differ from control plants during the first dry season, but supported fewer mines than both control and JA‐elicited plants during the wet season and the second dry season. Sucking insects (excluding whiteflies) exhibited a significant season × treatment interaction, with higher abundance on control plants during the first dry season compared to SA‐ and JA‐elicited plants, but greater abundance on JA‐elicited plants during the wet season (*χ* ^2^_4_ = 19.46, *p* < 0.001, Figure 7D). Whitefly abundance was also influenced by the season × treatment interaction (*χ* ^2^_4_ = 85.62, *p* < 0.001, Figure 7D). During the first dry season, JA‐elicited plants harboured a 0.36‐fold reduction in whiteflies compared to SA‐elicited and control plants. In contrast, during the wet season and second dry season, whitefly abundance was reduced by 63% and 74%, respectively, on SA‐elicited plants compared to JA‐elicited plants. Among carnivores, treatment effects were weaker and less temporally structured. Among the most abundant carnivorous taxa, Clubionidae spiders and *Macrolophus aragarcanus* showed significant seasonal variation (*χ* ^2^_2_ = 10.74, *p* = 0.004; *χ* ^2^_2_ = 12.34, *p* = 0.002, respectively), whereas their abundance did not differ among plant defence treatments (Table S1). Clubionidae spiders were most abundant in the wet season (estimated marginal mean ± SE = 0.0255 ± 0.0087), with significantly lower abundance in the second dry season (0.0085 ± 0.0034) and near‐zero abundance in the first dry season, while *M. aragarcanus* peaked in the second dry season (Figure 7). In contrast, the abundance of Nabidae damsel bugs and *Peucetia rubrolineata* lynx spiders did not vary across seasons or treatments (Table S1). Together, these results demonstrate that the effects of plant defences on arthropod communities are strongly time‐dependent, with the magnitude and direction of responses varying across functional groups and emerging most clearly during dry season conditions.

**FIGURE 6:**
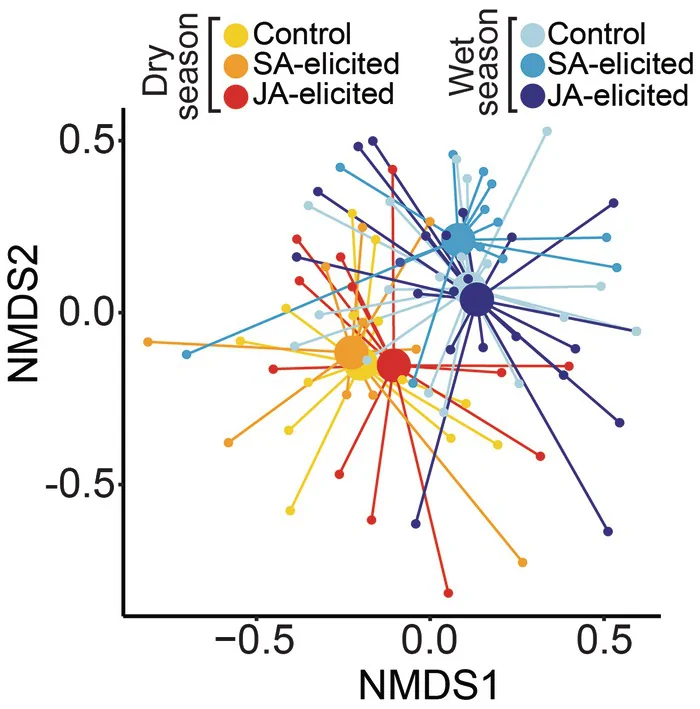
Non‐metric multidimensional scaling (NMDS) spider plot of arthropod community composition associated with *T. adenantha* plants subjected to different defence elicitation treatments across dry and wet seasons. Lines connect individual samples to treatment centroids (mean community composition). Ordination is based on Bray–Curtis dissimilarities of arthropod abundance. Plant defence treatments are indicated by colour: Untreated control plants (yellow and light blue symbols and lines), salicylic acid–elicited plants (SA; orange and medium blue) and jasmonic acid–elicited plants (JA; red and navy blue). Warm colours denote dry season samples, whereas cool colours denote wet‐season samples.

**FIGURE 7:**
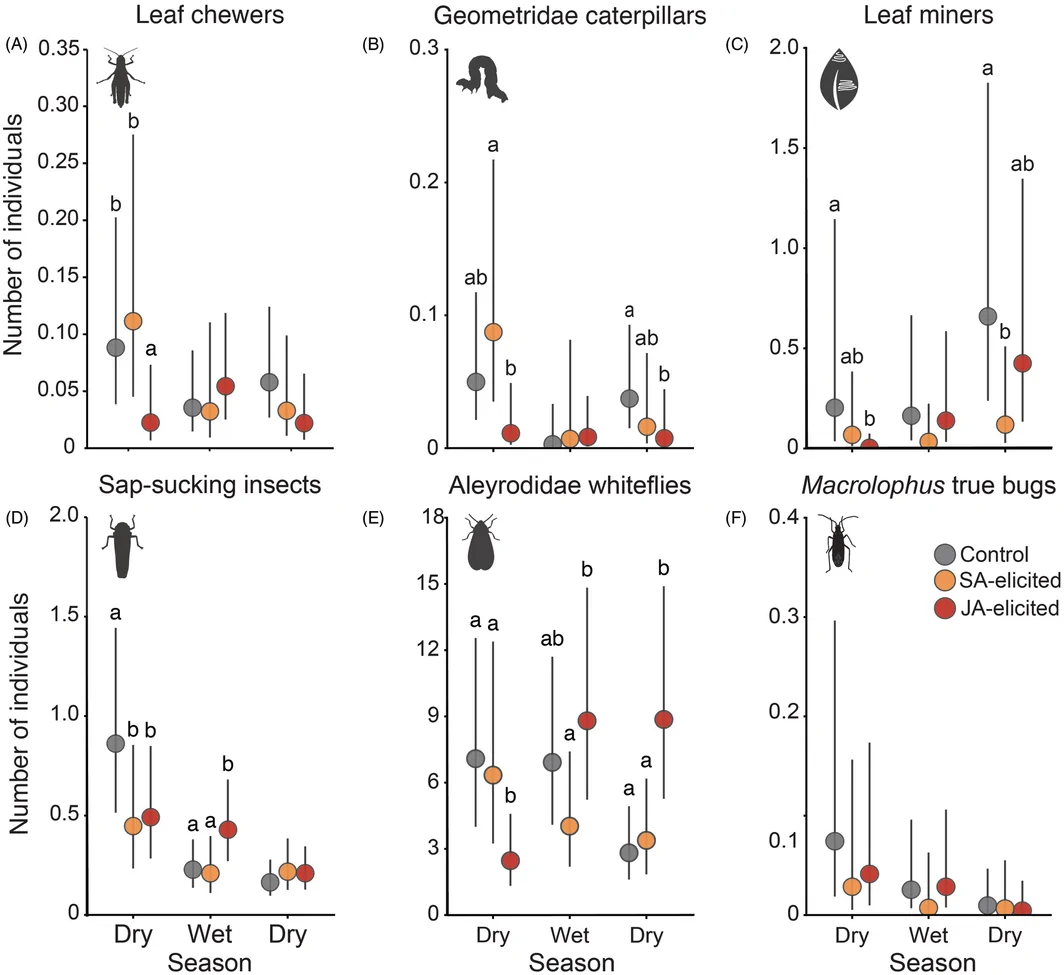
Seasonal variation in arthropod abundance in response to plant defence elicitation in *T. adenantha*. Panels show back‐transformed estimated marginal means (±95% confidence intervals) of (A) leaf chewers, (B) Geometridae sp. caterpillars, (C) leaf miners, (D) sap‐sucking insects (excluding whiteflies) (E) Aleyrodidae whiteflies, and (F) *Macrolophus* true bugs recorded throughout the experiment and separated by season. Plant defence treatments are represented by colour: Untreated control plants (grey symbols), salicylic acid–elicited plants (SA; orange symbols), and jasmonic acid–elicited plants (JA; red symbols). Different letters indicate significant differences among treatments within each season (*p* < 0.05, post hoc tests). [Correction added on 15 July 2026, after first online publication:The spelling of “individuals” in Figure 7(a) has been corrected.]

### 3.6 Plant reproductive output and seed damage

JA‐elicited plants produced nearly three times more flowerheads than control plants (*χ* ^2^_2_ = 12.00, *p* = 0.003, Figure 8A), but the number of ovaries per flowerhead was not affected (*χ* ^2^_2_ = 0.45, *p* = 0.647). Elicitation of plant defences reduced the probability of ovary fertilization, resulting in a 41% decrease relative to control plants (*χ* ^2^_2_ = 131.24, *p* < 0.001, Figure 8B). Although the probability of fertilization per flowerhead was lower in JA‐elicited plants, the total number of undamaged seeds per plant, our proxy for plant fecundity, did not differ between JA‐elicited and control plants (*χ* ^2^_2_ = 11.52, *p* = 0.003, Figure 8C). In contrast, SA‐elicited plants produced 0.62‐fold fewer seeds compared to JA‐elicited and control plants. Flowerhead damage strongly reduced seed production. Damaged flowerheads exhibited a 49% reduction in the probability of seed set (undamaged, fertilized ovaries) compared to undamaged flowerheads (*χ* ^2^_1_ = 52.08, *p* < 0.001, Figure 8D), indicating that pre‐dispersal seed predation directly reduced short‐term fecundity. Both JA and SA elicitation reduced the probability of ovary damage by 0.78‐fold relative to control plants (*χ* ^2^_2_ = 21.67, *p* < 0.001, Figure 8E). Each *Trupanea* larva showed a lower probability of ovary consumption on JA‐elicited plants, consuming 17% fewer ovaries than on control plants, and 26% fewer ovaries than on SA‐elicited plants (*χ* ^2^_2_ = 17.97, *p* < 0.001, Figure 8F). Within the guild of pre‐dispersal seed predators, JA elicitation reduced the abundance of *Trupanea* sp. (*χ* ^2^_2_ = 12.48, *p* = 0.002, Figure 8G), while increasing the abundance of *Melanagromyza* sp. (*χ* ^2^_2_ = 8.49, *p* = 0.014, Figure 8G) and the damage caused by Geometridae sp. compared to control plants (*χ* ^2^_2_ = 7.10, *p* = 0.029, Figure 8G). The probability of *Trupanea* parasitism did not differ among treatments (*χ* ^2^_2_ = 1.66, *p* = 0.435).

**FIGURE 8:**
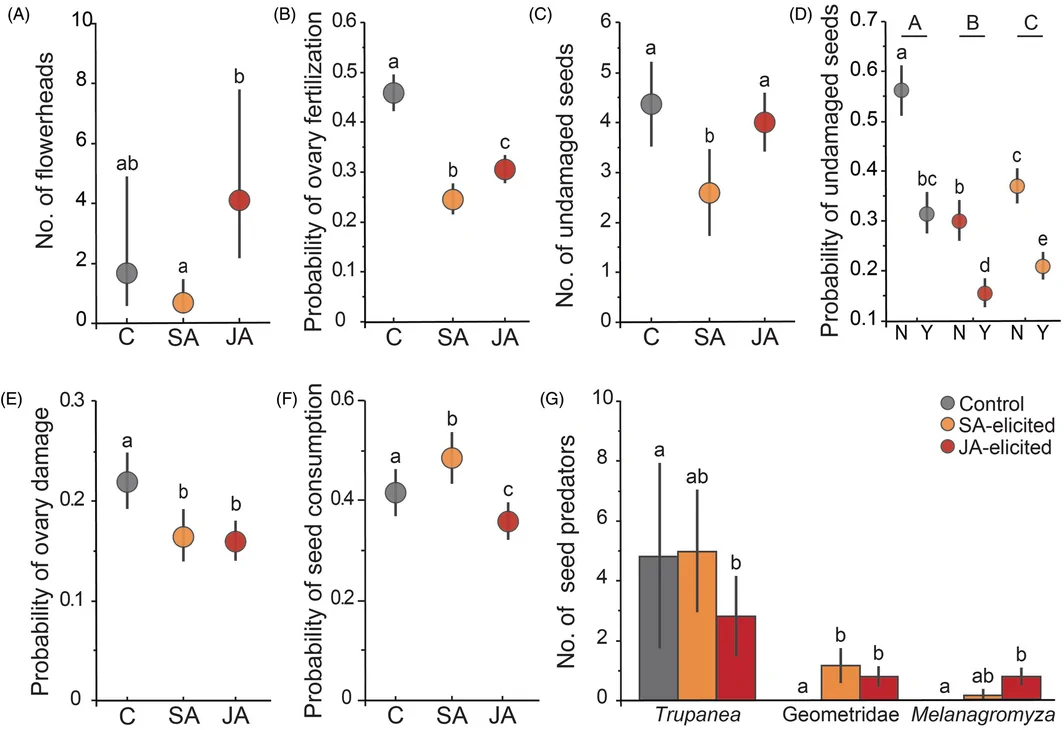
Effects of plant defence elicitation on short‐term fecundity and pre‐dispersal seed predation in *T. adenantha*. (A) number of flowerheads per plant, (B) probability of ovary fertilization, (C) total number of undamaged seeds per plant, (D) Probability of producing undamaged seeds in infested flowerheads (N = no; Y = yes) (E) probability of ovary damage, (F) probability of seed consumption by *Trupanea* sp., and (G) mean abundance (±1 SE) of the most common pre‐dispersal seed predator taxa. Plant defence treatments are indicated by colour: Untreated control plants (grey), salicylic acid–elicited plants (SA; orange) and jasmonic acid–elicited plants (JA; red). For panels (A–F), points represent back‐transformed estimated marginal means and error bars indicate 95% confidence intervals. Different letters indicate significant differences among treatments (*p* < 0.05, post hoc tests).

## 4 DISCUSSION

Trade‐offs among defence, growth and reproduction lie at the core of plant defence theory, yet how phytohormone signalling mediates these trade‐offs in natural systems remains poorly resolved. Most existing work derives from short‐term, controlled experiments, limiting predictions under seasonally variable and ecologically realistic conditions, particularly in tropical systems, where perennial plants sustain continuous biotic interactions. Here, we provide a rare field‐based test of defence–growth–reproduction trade‐offs by linking phytohormone elicitation to arthropod community assembly, plant traits and short‐term fecundity (Figure 9). Jasmonic acid (JA)‐ and salicylic acid (SA)‐elicited plants hosted distinct arthropod assemblages, with differences strongest in the dry season, when growth and insect abundance decline, revealing a pronounced temporal dimension to defence outcomes. Our results show that JA and SA signalling operate not as a simple dichotomy but as an interconnected signalling network with overlapping effects. Remarkably, both JA and SA elicitation imposed reproductive costs, either by disrupting pollination or diverting resources from seed production. Together, our findings show that context‐dependent defence signalling restructures multitrophic interactions, ecological communities and reproductive success, advancing a predictive framework for plant defence theory.

**FIGURE 9:**
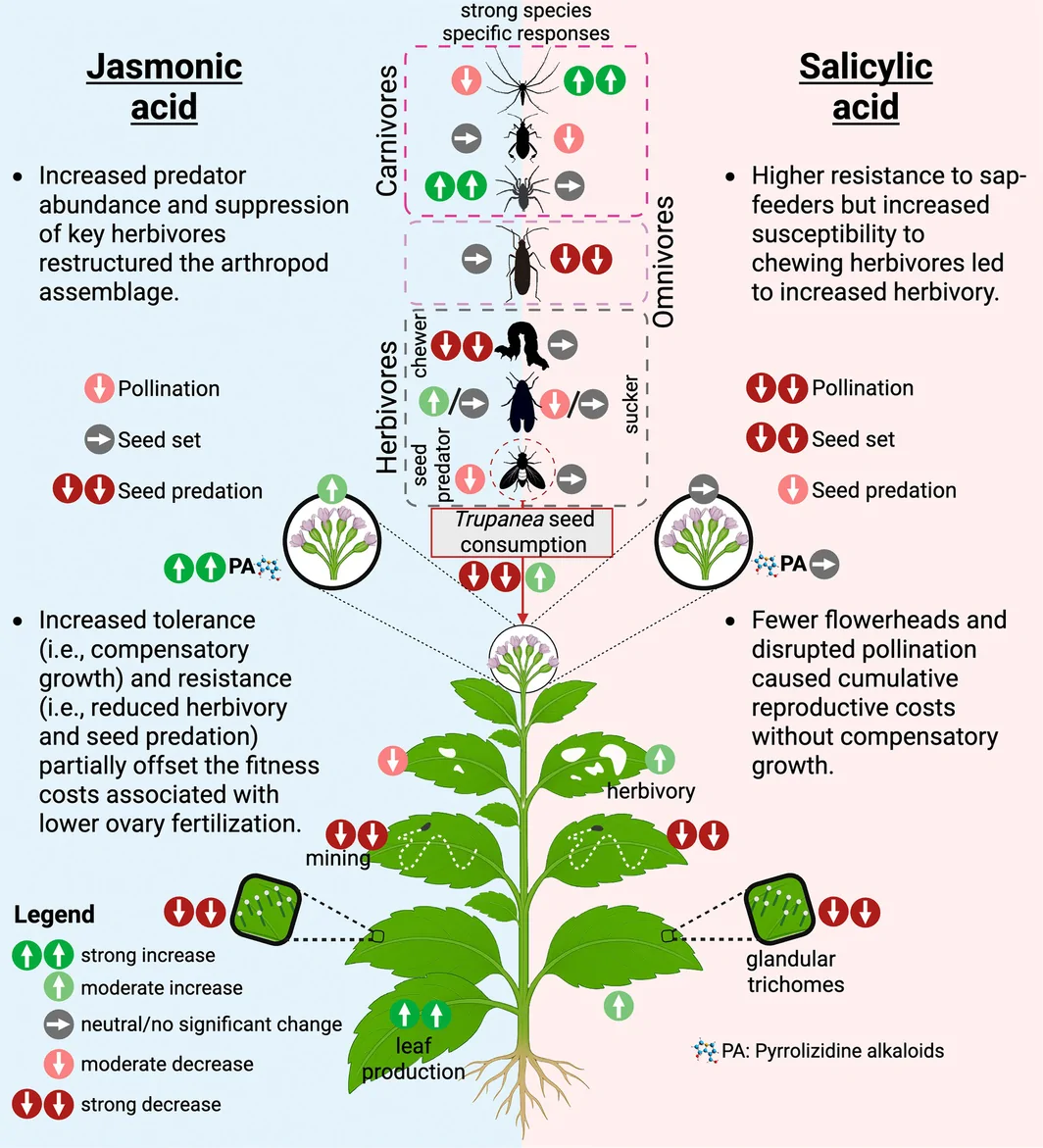
Schematic summary of key results. Defence elicitation treatments are shown in comparison to control plants, highlighting differences in arthropod responses and plant traits. Created in BioRender.

### 4.1 Consequences of defences on herbivory, feeding guilds and species‐specific responses

Our results show that manipulating phytohormone signalling reshapes species interactions and community dynamics by altering plant defensive traits, underscoring the importance of multi‐guild experiments for revealing ecological trade‐offs of defence elicitation. Prior work shows that JA activation typically increases resistance to chewing herbivores, whereas SA signalling often reduces sap‐feeder abundance or damage (Kessler & Kalske, 2018; Schweiger et al., 2014). Consistent with these patterns, in our study, JA‐elicited plants experienced less foliar damage and seed predation than control plants, reflecting downstream effects of induced defences. In contrast, SA‐elicited plants hosted fewer whiteflies, the most abundant herbivore, but suffered increased susceptibility to both foliar chewers and seed predators compared to JA‐elicited plants, suggesting weaker protection against these guilds. We attributed the reduced attack by pre‐dispersal seed predators and lower chewing herbivore abundance on JA‐treated plants to increased production of pyrrolizidine alkaloids, secondary metabolites regulated by the jasmonic acid pathway (Chen et al., 2019; Schramm et al., 2019). Notably, we did not observe taxa known to sequester pyrrolizidine alkaloids, and the sequestration capacity of the herbivores recorded here remains unknown.

Despite significant effects on herbivore composition and damage, JA and SA elicitation did not alter omnivore or carnivore abundance at the guild level. This contrasts with systems where omnivores prefer control plants but respond weakly to herbivore prey that fed on induced versus uninduced plants (Thaler et al., 2015). Alternatively, interactions among herbivore guilds may have disrupted volatile cues that attract natural enemies, obscuring broader top‐down responses (Zhang et al., 2019). Species‐level analyses, however, revealed pronounced shifts. SA‐elicited plants supported more *Peucetia* lynx spiders but fewer Nabidae predators, whereas JA‐elicited plants attracted more Clubionidae spiders, patterns likely driven by shifts in prey availability and predator foraging strategies (Kersch‐Becker et al., 2018a; Wise, 2006). The dominant omnivore, the true bug *Macrolophus aragarcanus*, declined on SA‐elicited plants, potentially due to higher SA resistance, reduced prey, or increased predation (Hatherly et al., 2009; Lins et al., 2014). These responses may also be influenced by the reduced glandular trichome density observed on elicited plants, which likely altered plant surface structure and predator–prey encounter rates. Such physical changes can differentially benefit species with distinct foraging modes or mobility. In addition, leaf miners also declined on both JA‐ and SA‐elicited plants, consistent with reduced colonization or larval survival following defence induction (Black et al., 2003; Hopkins & Memmott, 2003). These results demonstrate the importance of integrating guild‐ and species‐level perspectives: while guild analyses suggest limited effects, species‐level responses reveal how phytohormone signalling structures arthropod communities across trophic levels.

### 4.2 Temporal effects of plant defence induction

The arthropod community associated with *T. adenantha* exhibited strong seasonal variation that varied with plant defence elicitation. Arthropod abundance and species composition shifted markedly across seasons, peaking in the wet season, when increased vegetative and reproductive growth likely enhanced resource availability and habitat complexity (Richards & Coley, 2007; Romero & Vasconcellos‐Neto, 2003, 2005). This strong seasonal signal emphasizes the role of phenological context in mediating how plant defences shape community dynamics, particularly in perennial tropical systems (Cosmo et al., 2026). Plant defence elicitation altered herbivore pressure in a season‐dependent manner. Within this seasonal framework, defence elicitation modified herbivore pressure in a context‐dependent manner. JA‐elicited plants consistently supported fewer chewing herbivores during the dry seasons, when leaf production is limited and herbivore pressure is concentrated on existing tissues. This pattern highlights the effectiveness of JA‐mediated defences under conditions of resource scarcity, when the fitness costs of herbivory may be greatest (Aide, 1992; Cosmo et al., 2019). In contrast, leaf miners declined on both JA‐ and SA‐elicited plants across seasons, suggesting that induction of either pathway can suppress this guild. However, the underlying mechanisms likely differ, with JA acting through direct resistance and SA acting through indirect, enemy‐mediated effects (Hopkins & Memmott, 2003; Karban, 1993).

Whiteflies and predators exhibited similarly dynamic responses. Whiteflies were initially less abundant on JA‐elicited plants, but their densities later surged during the wet season and remained high into the second dry season. At this peak, however, SA‐elicited plants supported significantly fewer whiteflies, suggesting that salicylate signalling may confer longer‐term suppression. Whiteflies can suppress JA defences, alter plant volatile profiles and recruit ant mutualists, enhancing their survival and feeding success (de Jesus et al., 2016; Zarate et al., 2007; Zhang et al., 2019). Predator responses were taxon‐specific: Clubionidae spiders were more abundant in the wet season, likely tracking higher habitat complexity and prey availability, whereas Nabidae damsel bugs and *Peucetia* lynx spiders showed no consistent treatment or seasonal effects. *M. aragarcanus*, however, was most abundant in the first dry season. These mixed responses suggest that prey availability, volatile cues, or intraguild interactions may influence higher trophic levels, emphasizing the need for experiments that manipulate both prey and defence signalling across seasons to disentangle direct and prey‐mediated effects. Together, these results indicate that the ecological outcomes of defence signalling depend not only on the defence pathway activated but also on seasonal variation in plant growth and arthropod life histories.

### 4.3 Phytohormone signalling shapes physical and chemical defences

Plants deploy both direct defences, such as trichomes and alkaloids, and indirect defences that recruit natural enemies. In our study, elicitation of both JA‐ and SA‐mediated defences suppressed glandular trichome production. This result contrasts with *Arabidopsis*, where JA increased trichome density, but aligns with evidence that SA can inhibit trichome development (Traw & Bergelson, 2003). Although we did not measure trichome stickiness, phytohormone elicitation may have altered trichome exudation or chemistry rather than density, as shown in tobacco following mechanical damage (Karban et al., 2019). Together, these findings suggest that phytohormone effects on structural defences can be highly context‐ and species‐dependent, particularly in non‐model, long‐lived perennials. In *T. adenantha*, reduced trichome density under both pathways may reflect resource limitation in young plants or regulatory constraints within the signalling networks, whereby investment in chemical defences trades off with physical structures (Agren & Schemske, 1993; Ballhorn et al., 2008, 2013). Because JA and SA also regulate development, defence elicitation may directly constrain trichome formation via hormonal effects on leaf development.

Despite their contrasting ecological effects, JA and SA elicitation reduced trichome density, but only JA consistently increased pyrrolizidine alkaloid (PA) concentrations, indicating divergence rather than full convergence in downstream chemical defences. Although both signalling pathways altered plant traits, similar changes in structural defences did not translate into equivalent ecological outcomes. For example, lynx spiders were more abundant on SA‐elicited plants despite trichome reductions, indicating that unmeasured traits, such as volatile organic compounds, plant quality, or prey accessibility, likely mediate predator responses. These results show that partial overlap defence expression can mask divergent ecological consequences, consistent with evidence that JA and SA signalling drive distinct, pathway‐specific metabolomic responses with contrasting effects on herbivory (Wei et al., 2021).

Patterns of pyrrolizidine alkaloid (PA) allocation were consistent with Optimal Defence Theory, with reproductive tissue and young leaves exhibiting higher PA concentration than mature and senescent leaves. JA elicitation further increased PA concentrations in reproductive tissues, corroborating previous findings that link jasmonate signalling to alkaloid biosynthesis (Chen et al., 2019). Notably, this JA elicitation increase was pronounced in flowerheads but not in leaves, suggesting that MeJA application may have promoted the reallocation of existing alkaloids to reproductive tissues rather than de novo synthesis (Wei et al., 2019). In contrast, SA had limited or no effect on PA levels in most tissues. Higher PA concentrations in flowerheads may deter endophagous herbivores that oviposit before floret maturation, a critical defence given the strong effects of flowerhead infestation on pollination success (Kersch‐Becker et al., 2018b; Romero et al., 2008; Romero & Vasconcellos‐Neto, 2004). Conversely, the lack of PA induction in SA‐elicited plants may have contributed to their increased susceptibility to damage by pre‐dispersal seed predators.

### 4.4 Defence signalling mediates growth–reproduction trade‐offs

We experimentally manipulated JA and SA signalling continuously for up to 16 months, allowing us to test how these pathways mediate growth–defence–reproduction trade‐offs. We define growth costs as trade‐offs between vegetative growth (leaf production) and reproductive investment (flower and seed production). JA‐elicited plants produced more leaves and flowerheads compared to control plants, indicating greater allocation to vegetative and reproductive structures. Although JA is often linked to reduced leaf expansion and accelerated senescence, these effects vary across species, resource availability and hormonal crosstalk (Chen et al., 2019; Gasperini & Howe, 2024; Schweiger et al., 2014). The absence of growth suppression and increased flowerhead production in JA‐elicited plants suggests a tolerance‐based strategy, such as compensatory responses or resource reallocation, rather than a simple cost of defence.

Both elicitation treatments reduced pollination success, resulting in a lower probability of ovary fertilization, even in JA‐elicited plants that produced more flowerheads. This pattern reveals a trade‐off between defence or growth investment and reproductive success, rather than a uniform decline in performance. Although some studies report no reproductive costs of induced defences (Agrawal, 1998, 1999), broader evidence shows that induction often reduces seed set, potentially due to altered floral traits or pollinator behaviour (Lucas‐Barbosa, 2016; Lucas‐Barbosa et al., 2011; Rusman et al., 2022; Strauss et al., 1996). Because JA and SA also regulate floral development, defence elicitation may directly affect flowers, including premature abortion (Luo et al., 2022; Yuan & Zhang, 2015). In *T. adenantha*, herbivores and predators may further reduce pollination by deterring pollinators in this obligate outcrossing species. Herbivory can alter pollinator visitation and network structure, even in the absence of changes in floral attractiveness (Lucas‐Barbosa et al., 2011; Rezende et al., 2025; Rusman et al., 2018).

Despite reduced pollination, JA‐elicited plants produced as many seeds as control plants, suggesting that growth–reproduction trade‐offs were offset by increased flowerhead production and reduced seed predation. In contrast, SA‐elicited plants experienced compounded fitness costs: lower flowerhead production, reduced fertilization and higher seed predation together reduced undamaged seed output. Differences in predator communities may have contributed to these outcomes, because the presence of spiders can deter pollinators from visiting flowers (Kersch‐Becker et al., 2018a; Romero & Koricheva, 2011). Together, these results show that JA and SA signalling generate distinct growth–reproduction trade‐offs with contrasting consequences for plant fitness.

## 5 CONCLUSIONS

We investigated the seasonal arthropod dynamics on a perennial tropical plant to assess how jasmonic acid (JA) and salicylic acid (SA) signalling shape herbivore colonization, predator associations and plant fecundity. Year‐long signalling manipulation revealed that JA and SA differentially structured arthropod guilds across seasons, altering community composition and trophic interactions. While defence elicitation reduced seed predation by limiting oviposition and herbivore success, it also imposed substantial reproductive costs, most notably through reduced pollination and seed set. These outcomes illustrate the complex ecological consequences of phytohormone‐mediated defences. JA‐elicited plants exhibited tolerance strategies, including compensatory growth and reduced seed predation, which partially offset reproductive costs. In contrast, SA‐elicited plants suffered compounded costs driven by higher herbivory, reduced flower production, increased seed predation, and shifts in biotic interactions that likely disrupted pollination. Together, these findings reveal that defence activation does not consistently enhance plant reproductive output and can generate trade‐offs that restructure ecological networks. By linking defence signalling to multitrophic outcomes across seasons, our study highlights the importance of evaluating both direct and indirect costs of defence, particularly in perennial species, and advances a more integrative understanding of how plant defence structures ecological community dynamics.

## AUTHOR CONTRIBUTIONS

Mônica F. Kersch‐Becker, Martin Pareja and Gustavo Q. Romero conceptualized the research. Mônica F. Kersch‐Becker, Guilherme P. Pinheiro, Thaís S. Schweller and José R. Trigo collected the data. Mônica F. Kersch‐Becker conducted data analysis and led the writing of the manuscript. All authors contributed critically to the drafts and gave final approval for publication.

## CONFLICT OF INTEREST STATEMENT

The authors declare there are no conflicts of interest.

## Supporting information


**Supporting Information S1.** Pyrrolizidine alkaloid content detailed methodology.

**Table S1.** Response and explanatory variables, model terms, chi‐square (*χ* ^2^) or *F*‐values, and significance levels (*p*) for plant attributes and arthropod responses to defence treatments. Significance levels are indicated as ***p < 0.001, **p < 0.01, *p < 0.05 and (*) 0.05 < *p* < 0.10. Response variables are grouped into categories: plant growth, phenology and herbivory; cumulative arthropod abundance; temporal arthropod dynamics; plant reproductive output and seed damage; and chemical and physical defences. Analyses of plant traits, plant reproductive output and pre‐dispersal seed predators, and chemical and physical defences were conducted in JMP 18, whereas analyses of cumulative and temporal arthropod abundance were conducted in R version 4.5.1 (2025‐06‐13).

## Data Availability

Data available in the Dryad Digital Repository at https://doi.org/10.5061/dryad.s7h44j1pf (Kersch‐Becker et al., 2026).
