# Supplementary material for: Complex multitrophic species interactions and fitness costs: Intricate consequences of jasmonate and salicylate induced plant defences
Source: J Anim Ecol. 2026 Apr 14;95(10):1859–75. doi: 10.1111/1365-2656.70243 (PMC13643180; doi:10.1111/1365-2656.70243)
Supplement: Supplementary file 1 [file JANE-95-1859-s001.docx]

***Supplementary Information 1***

***Pyrrolizidine alkaloid content.*** Five days after treatment application, we sampled 24 plants (8 replicates per treatment) and collected ca. 300 mg of senescent, mature and young leaves, as well as inflorescence buds and open inflorescences, to assess pyrrolizidine alkaloid (PA) content. To extract PAs, we freeze-dried the plant material for 48 h using a Labconco FreeZone 6 Liter Freeze Dry System. After drying, we weighed the samples and transferred them to test tubes (1.3 cm diameter × 10 cm height) containing 1 mL of ethanol for homogenization. We centrifuged the samples at 3000 rpm for 10 minutes. We removed the ethanolic layer and repeated the procedure two additional times. We combined the three ethanolic layers, adjusting the final volume to 5 mL with ethanol. We quantified PAs using a colorimetric method (Brown, 1984, 1985; Trigo et al., 1993). We transferred 1.5 to 4 mL aliquots of the ethanolic extract (depending on tissue type) to glass vials and evaporated them in a 90°C hot bath. We then added 500 µL of oxidation reagent (1 mL H₂O₂, 5 mg tetrasodium pyrophosphate, 200 mL MeOH), heated the samples at 100°C for 25 minutes, and allowed them to cool to room temperature. Next, we added 1 mL of isoamyl acetate and 0.1 mL of acetic anhydride, incubated the mixture at 100°C in a hot bath for another 25 minutes, and cooled the samples again. We added 1 mL of freshly prepared Ehrlich reagent (5.1 mL BF₃, 46.5 mL EtOH, 0.9 g dimethylaminobenzaldehyde), sealed the tubes, and incubated them at 60°C for 5 minutes in a hot bath. After cooling, we added 1 mL of acetone to bring the final volume to 4 mL. We measured absorbance at 561 nm using a SP-22 digital spectrophotometer (Biospectro; range: 325–1000 nm; resolution: ±0.078 A), performing readings in triplicate whenever possible. We quantified PAs by converting mean absorbance values to micrograms based on a calibration curve generated using the PA monocrotaline as a standard. We assumed that molar extinction coefficients did not differ among PA types. Final PA concentrations are expressed as μg per gram of dry weight tissue.
