# Supplementary material for: Complex multitrophic species interactions and fitness costs: Intricate consequences of jasmonate and salicylate induced plant defences
Source: J Anim Ecol. 2026 Apr 14;95(10):1859–75. doi: 10.1111/1365-2656.70243 (PMC13643180; doi:10.1111/1365-2656.70243)
Supplement: Supplementary file 2 [file JANE-95-1859-s002.docx]

Table S1. Response and explanatory variables, model terms, chi-square (χ^2^) or F-values, and significance levels (*P*) for plant attributes and arthropod responses to defense treatments. Significance levels are indicated as *** *P* < 0.001, ** *P* < 0.01, * *P* < 0.05, and (*) 0.05 < *P* < 0.10. Response variables are grouped into categories: plant growth, phenology and herbivory; cumulative arthropod abundance; temporal arthropod dynamics; reproductive output and seed damage; and chemical and physical defenses. Analyses of plant traits, plant reproductive output and pre-dispersal seed predators, and chemical and physical defenses were conducted in JMP 18, whereas analyses of cumulative and temporal arthropod abundance were conducted in R version 4.5.1 (2025-06-13).

| Response variables | Explanatory variable | Random effects | Model | | Family | | χ^2^ or F-values | | Prob> χ^2^ or Prob> F | |
| --- | --- | --- | --- | --- | --- | --- | --- | --- | --- | --- |
| **Plant growth, phenology and herbivory** | | | | | | | | | | |
| Herbivory level (arcsine) | Defense treatment  Trial  No. days alive | Block | GLMM | | Gamma | | 7.110_2_  34.741_2_  96.131_1_ | | 0.002**  <0.001***  <0.001*** | |
| Height (log) | Defense treatment  Initial Height  Trial  No. days alive | Block | GLMM | | Gaussian | | 0.231_2_  25.539_2_  5.208_2_  6.539_1_ | | 0.795  <0.001***  0.014*  0.013* | |
| Cumulative number of lost leaves | Defense treatment  Trial  No. days alive | Block | GLMM | | Negative binomial | | 4.290_2_  51.115_2_  101.260_1_ | | 0.020*  <0.001***  <0.001*** | |
| Cumulative number of leaves produced | Defense treatment  Trial  No. days alive | Block | GLMM | | Negative binomial | | 4.573_2_  61.192_2_  137.298_1_ | | 0.017*  <0.001***  <0.001*** | |
| **Cumulative abundance of arthropods** | | | | | | | | | | |
| Arthropod richness | Defense treatment  Trial  No. days alive |  | GLM | | Negative binomial | | 0.995_2_  17.801  16.176_1_ | | 0.608  <0.001***  <0.001*** | |
| Shannon diversity index (H’) (log) | Defense treatment  No. days alive |  | GLM | | Gaussian | | 4.397_2_  5.257_1_ | | 0.092  <0.001*** | |
| Abundance of sap-sucking insects without whiteflies | Defense treatment  Trial  No. days alive |  | GLMM | | Negative binomial | | 0.259_2_  14.362_2_  19.890_1_ | | 0.878  <0.001***  <0.001*** | |
| Abundance of whiteflies | Defense treatment | Trial | GLMM | | Negative binomial | | 5.553_2_ | | 0.062(*) | |
| Abundance of mining insects | Defense treatment  Trial  No. days alive | Block | GLMM | | Zero-inflated Negative binomial | | 6.483_2_  0.228_2_  10.211_1_ | | 0.0391*  0.633  0.001** | |
| Abundance of leaf chewing insects | Defense treatment  Trial  No. days alive | Block | GLMM | | Poisson | | 7.041_2_  3.672_2_  6.968_1_ | | 0.030*  0.159  0.008 | |
| Abundance of Geometridae spp. | Defense treatment  Trial  No. days alive |  | GLM | | Negative binomial | | 7.004_2_  3.106_2_  1.632_1_ | | 0.030*  0.212  0.201 | |
| Abundance of omnivorous insects | Defense treatment  Trial |  | GLM | | Negative binomial | | 1.555_2_  15.630_2_ | | 0.460  <0.001*** | |
| Abundance of *Macrolophus aragarcanus* | Defense treatment  Trial  No. days alive |  | GLM | | Negative binomial | | 7.308_2_  12.542_2_  0.226_1_ | | 0.026*  0.001**  0.633 | |
| Abundance of carnivorous insects | Defense treatment  Trial  No. days alive | Block | GLMM | | Negative binomial | | 0.691_2_  14.102_2_  9.762_1_ | | 0.708  <0.001***  0.002** | |
| Abundance of *Misumenops argenteus* | Defense treatment  Trial  No. days alive | Block | GLMM | | Negative binomial | | 2.494_2_  1.031_1_  3.070_1_ | | 0.433  0.597  0.080 | |
| Abundance of *Peucetia rubrolineata* | Defense treatment  Trial  No. days alive |  | GLM | | Zero inflated negative binomial | | 9.269_2_  13.164_2_  1.973_1_ | | 0.010*  0.001**  0.160 | |
| Abundance of Clubionidae spp. | Defense treatment  Trial  No. days alive |  | GLM | | Zero inflated Poisson | | 6.378_2_  21.091_2_  1.434_1_ | | 0.041*  <0.001***  0.231 | |
| Abundance of Nabidae spp. | Defense treatment  Trial  No. days alive | Block | GLMM | | Zero inflated Poisson | | 8.242_2_  1.665_2_  0.253_1_ | | 0.016*  0.435  0.615 | |
| **Temporal arthropod dynamics** | | | | | | | | | | |
| Abundance of arthropods (whiteflies excluded) | Defense treatment  Season  Defense treatment*season  Trial | Plant ID | | GLMM | | Negative binomial | | 1.064 _2_  3.636_2_  19.327_4_  7.576_2_ | | 0.587  0.162  <0.001*** 0.022* |
| Abundance of leaf chewing insects | Defense treatment  Season  Defense treatment*season  Trial | Plant ID | | GLMM | | Negative binomial | | 4.234_2_  2.964_2_  12.835_4_  0.476_2_ | | 0.120  0.227  0.012*  0.788 |
| Abundance of leaf mining insects | Defense treatment  Season  Defense treatment*season  Trial | Plant ID | | GLMM | | Zero inflated negative binomial | | 7.104_2_  23.400_2_  13.469_4_  6.978_2_ | | 0.029*  <0.001***  0.009**  0.031* |
| Abundance of Geometridae caterpillar | Defense treatment  Season  Defense treatment*season | Plant ID, trial | | GLMM | | Negative binomial | | 2.532_2_  11.851_2_  8.033_4_ | | 0.281  0.002**  0.090(*) |
| Abundance of sucking insects (whiteflies excluded) | Defense treatment  Season  Defense treatment*season  Trial | Plant ID | | GLMM | | Negative binomial | | 1.268_2_  32.618_2_  19.464_4_  37.842_2_ | | 0.530  <0.001***  <0.001***  <0.001*** |
| Abundance of whiteflies | Defense treatment  Season  Defense treatment*season  Trial | Plant ID | | GLMM | | Negative binomial | | 0.790_2_  23.254_2_  85.622_4_  47.152_2_ | | 0.674  <0.001***  <0.001***  <0.001*** |
| Abundance of *Macrolophus aragarcanus* | Defense treatment  Season  Defense treatment*season  Trial | Plant ID | | GLMM | | Negative binomial | | 2.327_2_  12.338_2_  2.547_4_  3.328_2_ | | 0.312  0.002**  0.636  0.189 |
| Carnivore abundance | Defense treatment  Season  Defense treatment*season  Trial | Plant ID | | GLMM | | Negative binomial | | 0.326_2_  27.580_2_  2.568_4_  26.378_2_ | | 0.312  <0.001*** <0.001***  0.189 |
| Abundance of Clubionidae spp. | Defense treatment  Season  Defense treatment*season  Trial | Plant ID | | GLMM | | Negative binomial | | 0.000_2_  10.748_2_  0.854_4_  35.914_2_ | | 1.000  0.004**  0.931  <0.001*** |
| Abundance of Nabidae spp. | Defense treatment  Season  Defense treatment*season | Plant ID | | GLMM | | Negative binomial | | 0.000_2_  1.934_2_  2.767_4_ | | 1.000  0.371  0.598 |
| Abundance of *Peucetia rubrolineata* | Defense treatment  Season  Defense treatment*season  Trial | Plant ID | | GLMM | | Negative binomial | | 0.000_2_  0.000_2_  4.283_4_  2.247_2_ | | 1.000  1.000  0.369  0.325 |
| **Plant reproductive output and pre-dispersal seed predators** | | | | | | | | | | |
| No. of flowerheads | Defense treatment  Trial  No. days alive |  | | GLM | | Negative binomial | | 12.000_2_  37.428_2_  9.306_1_ | | 0.003**  <0.001***  0.0023** |
| No. of ovaries | Defense treatment  Trial |  | | GLM | | Gaussian | | 0.446  1.054 | | 0.647  0.369 |
| Probability of ovary fertilization | Defense treatment  Trial |  | | GLM | | Binomial | | 131.239_2_  69.175_2_ | | <0.001***  <0.001*** |
| No. of undamaged seeds | Defense treatment  Trial  No. days alive |  | | GLM | | Zero inflated negative binomial | | 11.524_2_  57.971_2_  29.375_1_ | | 0.003**  <0.001***  <0.001*** |
| Probability of damaged ovaries | Defense treatment  Trial |  | | GLM | | Binomial | | 21.669_2_  41.533_2_ | | <0.001***  <0.001*** |
| Probability of seed consumption by *Trupanea* | Defense treatment  No. of *Trupanea* (log) |  | | GLM | | Binomial | | 17.967_2_  0.665_1_ | | <0.001***  0.415 |
| Probability of undamaged seeds | Defense treatment  Infestation  Defense treatment*Infestation  Trial |  | | GLM | | Binomial | | 134.193_2_  190.429_1_  2.266_2_  94.143_2_ | | <0.001***  <0.001***  0.312  <0.001*** |
| Abundance of *Trupanea* | Defense treatment  No. of flowerheads |  | | GLM | | Poisson | | 8.486_2_  3.996_1_ | | 0.002**  <0.001*** |
| Abundance of Geometridae | Defense treatment  No. of flowerheads |  | | GLM | | Poisson | | 7.104_2_  13.878_1_ | | 0.029*  <0.001*** |
| Abundance of *Melanagromyza* | Defense treatment  No. of flowerheads |  | | GLM | | Poisson | | 8.486_2_  3.996_1_ | | 0.014*  0.046* |
| Probability of *Trupanea* parasitism | Defense treatment  No. of flowerheads |  | | GLM | | Binomial | | 1.664_2_  1.667_1_ | | 0.435  0.197 |
| **Chemical and physical defenses** | | | | | | | | | | |
| Pyrrolizidine alkaloid content in flower buds | Defense treatment  No. of leaves |  | GLM | | Cauchy | | 7.968_2_  1.694_1_ | | 0.019*  0.193 | |
| Pyrrolizidine alkaloid content in open flowerheads | Defense treatment  No. of leaves |  | GLM | | Cauchy | | 15.097_2_  9.332_1_ | | <0.001***  0.002** | |
| Pyrrolizidine alkaloid content in young leaves | Defense treatment  No. of leaves |  | GLM | | Cauchy | | 0.150_2_  22.578_1_ | | 0.928  <0.001*** | |
| Pyrrolizidine alkaloid content in mature leaves | Defense treatment  No. of leaves |  | GLM | | Cauchy | | 40.584_2_  29.335_1_ | | <0.001***  <0.001*** | |
| Pyrrolizidine alkaloid content in senescent leaves | Defense treatment  No. of leaves |  | GLM | | Cauchy | | 6.068_2_  11.167_1_ | | 0.048*  <0.001*** | |
| Glandular trichome density | Defense treatment |  | GLM | | Negative binomial | | 8.970_2_ | | 0.011* | |
| Non-glandular trichome density | Defense treatment |  | GLM | | Negative binomial | | 10.667_2_ | | 0.005** | |
